# Enhanced Apparent Oral Bioavailability of Berberine Through BioSOLVE Technology: Pharmacokinetic Evaluation of Metaberine™

**DOI:** 10.7759/cureus.109791

**Published:** 2026-05-28

**Authors:** Shankaranarayanan Jeyakodi, Arunkanth Krishnakumar, Kiran DA

**Affiliations:** 1 Research and Development, Zeus Hygia Life Sciences, Hyderabad, IND; 2 Research and Development, Apollo Biosciences, Bengaluru, IND

**Keywords:** berberine, bioavailability, biosolve technology, metaberine, pharmacokinetics

## Abstract

Background

Berberine is an isoquinoline alkaloid known for its potential role in supporting metabolic, cardiovascular, and gastrointestinal functions. Its clinical application, however, remains constrained by limited oral absorption, which is influenced by factors such as low solubility, restricted intestinal permeability, metabolism, and transport-mediated efflux.

Purpose

This study compared the oral pharmacokinetics of Metaberine™, a novel berberine formulation developed with BioSOLVE technology, against conventional berberine in male rats. The objective was to determine whether the formulation enhances peak plasma concentration (C_max_) and systemic exposure.

Methods

Male Sprague-Dawley rats were orally administered the test substance or conventional berberine at a dose of 200 mg/kg body weight. Blood samples were collected at predefined intervals and analyzed for plasma berberine concentrations using a validated liquid chromatography-tandem mass spectrometry (LC-MS/MS) method. Pharmacokinetic parameters, including area under the plasma concentration-time curve (AUC) from time 0 to 8 h (AUC_0-8_), 0 to 48 h (AUC_0-48_), and 0 to infinity (AUC_0-∞_), peak plasma concentration (C_max_), time to reach C_max_ (T_max_), apparent terminal half-life (t_1/2_), and mean residence time (MRT_(obs)_) were calculated by non-compartmental analysis.

Results

There were no treatment-related changes in clinical signs, morbidity, or mortality. The test substance demonstrated higher early systemic exposure and markedly increased peak plasma concentrations compared with conventional berberine. The AUC_0-8_ (177.7 ng·h/mL) of test substance was 2.6-fold higher, and C_max_ (139.26 ng/mL) was 11.3-fold higher than those of conventional berberine (68.28 ng·h/mL and 12.32 ng/mL, respectively). Log-transformed analysis showed a significant increase in AUC_0-8_ (geometric mean ratio (GMR): 2.07; 90% confidence interval (CI): 1.25-3.41; p < 0.05) and C_max_ (GMR: 6.46; 90% CI: 2.88-14.50; p < 0.05). The AUC_0-48_ and AUC_0-∞_ were not significantly different between groups. Considering the amount of active berberine, the test substance exhibited 8.6-fold higher AUC_0-8_, 5.5-fold higher AUC_0-48_, and 37.3-fold higher C_max_ per mg/kg active than conventional berberine. The test substance also exhibited a markedly earlier T_max_ (0.5 h vs. 8 h), indicating more rapid systemic appearance of berberine, along with a prolonged apparent terminal half-life (8.63 h vs. 3.64 h). However, T_max_ interpretation is limited by the absence of sampling prior to 0.5 h.

Conclusion

Overall, Metaberine™ enhanced the apparent oral bioavailability of berberine, primarily through increased early systemic exposure and peak plasma concentration. These findings suggest that the formulation developed using BioSOLVE technology alters the oral pharmacokinetics of berberine under the conditions of this study, supporting further evaluation in additional preclinical and clinical investigations.

## Introduction

Berberine is a benzylisoquinoline alkaloid. Berberine-containing plants were documented as early as 650 BC in Assyrian clay tablets [1] and have featured in ancient Chinese medical texts dating back to the Han Dynasty (25-220 AD) [2]. In Ayurveda, *Berberis* species were traditionally employed to treat various ailments, including indigestion; dysentery; ear, eye, and mouth infections; and hemorrhoids, and as an antidote for toxins [1]. Berberine has been derived from various plants, including *Berberis vulgaris* (barberry), *Coptis chinensis* (Chinese goldthread), and *Hydrastis canadensis* (goldenseal) [1]. Berberine was first isolated as a pure compound from *B. vulgaris* in 1830 [3]. Meta-analysis of clinical studies has demonstrated that berberine possesses multiple health benefits, including support for healthy blood lipid [4-12] and blood glucose levels [9,13-15], cardiovascular health [16-19], menstrual health [20], weight management [15,21-23], and gastrointestinal health [24].

Despite its broad therapeutic promise, berberine’s clinical utility is severely limited by its extremely low oral bioavailability, typically reported to be less than 1% in both animals and humans [25-27]. Berberine’s low oral bioavailability is primarily due to poor aqueous solubility and low intestinal permeability [26-29]; extensive first-pass metabolism in the intestine and liver mediated by cytochrome P450 enzymes and gut microbiota [25,27-32]; active efflux by P-glycoprotein (P-gp), which pumps berberine back into the intestinal lumen [27,28,30,31]; and its predominant hepatic distribution with rapid systemic elimination, resulting in low plasma concentrations despite high tissue levels [29,30,32]. Recent research has focused on understanding these barriers and developing novel formulation strategies such as nanoparticles [33-35], phytosomes [36], microemulsions [37], and solid dispersions [38]. Other delivery systems, such as bilosomes, proliposomes, and cremochylomicrons, target lymphatic absorption and bypass first-pass metabolism, with 2-6-fold increases in bioavailability [39,40]. Improved formulations have demonstrated enhanced glycemic control and organ protection [39,41-43]. Importantly, newer high-bioavailability formulations have shown no significant adverse effects in short-term human trials [44-46]. However, many of these systems involve complex manufacturing processes, specialized excipients, or administration of relatively high doses, including excipients, to achieve therapeutic effects. Conventional berberine supplementation typically requires daily doses of 0.5-1.5 g, which may limit tolerability and compliance. There remains a need for formulations capable of enhancing systemic exposure at substantially lower oral doses, typically at 200 mg/day, using safe, food-grade components suitable for long-term use.

Metaberine™ is a berberine formulation recommended at a dose of 200 mg/day developed using BioSOLVE technology, designed to promote rapid dispersion and systemic availability while maintaining compatibility with conventional oral dosage forms. BioSOLVE technology is a proprietary processing approach in which the herbal active ingredient is solubilized, homogenized to produce a uniform microparticulate system, and encapsulated within a food-grade polysaccharide matrix. This process enables complete and uniform encapsulation of the bioactive within an amphiphilic carrier system. Upon exposure to gastric fluid, the hydrophilic domains of the carrier promote rapid dispersion and apparent solubilization, forming a fine and uniform colloidal system. This may facilitate improved interaction with the intestinal environment and support enhanced absorption of the encapsulated bioactive. Collectively, these characteristics are expected to enhance solubility, absorption efficiency, and oral bioavailability of berberine at comparatively lower doses.

Advanced delivery systems are specifically designed to modify solubility, dissolution behavior, and intestinal absorption, and therefore, the formulation itself constitutes an integral component of the therapeutic intervention rather than merely a carrier of the active compound. Consequently, assessment of the complete formulation provides clinically relevant information regarding its ability to enhance systemic exposure compared with conventional preparations. Similar comparative approaches have been widely employed in pharmacokinetic (PK) studies evaluating nanoformulations, lipid-based systems, inclusion complexes, and other delivery technologies against free drug or conventional salt forms [44,46,47]. The present study thus aimed to evaluate the PK profile of this new formulation (Metaberine™) relative to conventional berberine hydrochloride following oral administration, with emphasis on its ability to enhance peak plasma concentration and apparent systemic exposure.

## Materials and methods

Materials

The test substance was obtained from Zeus Hygia Life Sciences Private Limited, Hyderabad. The formulation was designed to produce rapid aqueous dispersion and apparent solubilization of berberine upon exposure to gastrointestinal fluids, forming a fine colloidal system that enhances intestinal absorption. The comparator, conventional berberine (99% purity), was procured from Sigma-Aldrich, India, and carboxy methylcellulose (CMC) was obtained from Loba Chemie Private Limited, India.

Animal husbandry and care

Male Sprague-Dawley rats (7-8 weeks old, body weight of not less than 170 g) bred in-house (Apollo Biosciences, Bengaluru, India) were used. Animals were housed in polypropylene cages with stainless steel mesh tops, two animals per cage, with sterilized corn cob bedding (Rowan Agro Nature, India). The laboratory environment was maintained at 19°C-25°C, 30%-70% relative humidity, 12 h light/dark cycle, and 12-15 air changes per hour. Animals were fed ad libitum with VRK Scientific Choice Laboratory Animal Feed (M/S VRK Nutritional Solutions, India) and received potable water purified by reverse osmosis in polypropylene bottles with stainless steel sipper tubes. Animals were acclimatized for six days before the initiation of dosing. Veterinary inspections ensured only healthy animals were included. Body weight was recorded at the start of acclimatization and immediately prior to dosing. Animals were observed at least twice daily throughout the experimental period for mortality, morbidity, and clinical signs of distress or abnormal behavior. Additional observations were conducted during acclimatization to ensure suitability for inclusion in the study. These assessments were intended to monitor general health status following dosing. No hematological, biochemical, or histopathological evaluations were performed, as the study was designed as a single-dose PK investigation rather than a toxicological assessment. Tail marking with permanent markers [48] enabled identification during acclimatization, followed by body marking with picric acid [49] during dosing. Cages were labeled with study-specific information, including group, strain, sex, and experimental dates.

Dose formulation and administration

Distilled water was selected as the vehicle for the test substance based on the solubility profile of the substance, and 0.5% CMC was used as the vehicle for conventional berberine. Test formulations were freshly prepared daily, ensuring complete dissolution or suspension of the test item and homogenized by vortexing prior to administration. Dose preparations consisted of the test or conventional substance at a concentration of 20.3 mg/mL of vehicle to achieve a target dose of 200 mg/kg body weight at a dose volume of not more than 10 mL/kg. Dose volumes for individual animals were adjusted based on the recorded body weight that was obtained immediately prior to dosing. Oral administration was performed at similar timings using an oral intubation needle.

Experimental design and ethics

The PK study was conducted following the guidelines outlined in the Gazette of India (2018) and approved by the Institutional Animal Ethics Committee of Apollo Biosciences, Bengaluru, India (Approval protocol no. ABS-IAEC-133-2024-25). The study design and reporting were aligned with applicable principles of the Animal Research: Reporting of In Vivo Experiments (ARRIVE) guidelines [50]. Only animals with body weights within approximately ±20% of the group mean were included. Sixteen rats were then allocated to two groups (n = 8 per group) using body weight-stratified randomization to ensure comparable baseline body weights prior to dosing. The sample size (n = 8 per group) was considered sufficient to characterize PK profiles and detect formulation-related differences in exposure while adhering to ethical principles to minimize animal use. Blinding was not implemented because outcome measures consisted of objective analytical quantification of plasma concentrations. Group 1 received the test substance at a single oral dose of 200 mg/kg body weight, and group 2 received the conventional berberine at a single oral dose of 200 mg/kg body weight. Animals were fasted overnight prior to dosing to minimize food-related variability in absorption.

Blood collection and sample handling

Blood samples (0.1-0.2 mL) were collected from the retro-orbital plexus under isoflurane anesthesia [51] at 0 (pre-dose), 0.5, 1, 2, 4, 8, 12, 24, and 48 h post-dose. Samples were collected into heparinized microcentrifuge tubes and immediately centrifuged at 4,000 rpm for 10 minutes to separate plasma. The resulting plasma was transferred to labeled tubes and stored at -20°C until liquid chromatography-tandem mass spectrometry (LC-MS/MS) analysis. Animals were monitored twice daily for clinical signs, morbidity, and mortality throughout the study period.

LC-MS/MS analysis

Berberine quantification was performed by adopting a validated LC-MS/MS method [52,53], employing berberine chloride dihydrate as the reference standard and crizotinib as the internal standard (IS). Berberine had good linearity in the range of 2.0-1,600 ng·mL^-1^ with the lower limit of quantitation of 2.0 ng·mL^-1^. Intra- and inter-day accuracy ranged from 96.00% to 114.99%, with precision (%CV) below 10%. The mean extraction recovery of berberine from plasma was within ±15%. Each analytical batch included calibration standards and quality control samples to ensure assay performance. The assay quantified unchanged berberine; major metabolites were not measured. A stock solution of berberine (5 mg in 5 mL methanol) was serially diluted to prepare calibration standards. For sample preparation, 100 µL of plasma was spiked with 50 µL of IS dilution (500 ng/mL crizotinib) and vortexed. Liquid-liquid extraction was performed using 1.5 mL of ethyl acetate: n-hexane (70:30 v/v), followed by vortexing for 10 minutes and centrifugation. The resulting organic layer was collected, evaporated to dryness, and reconstituted in 300 µL of mobile phase prior to injection. Each plasma sample was analyzed in triplicate to ensure analytical reliability. Chromatographic separation was achieved using a Shimadzu LCMS-2040 system controlled by LabSolutions LCMS software (Shimadzu Corporation, Kyoto, Japan), equipped with a Phenomenex Luna Omega PS C18 column (50 × 4.6 mm, 5 µm) maintained at 40°C. The mobile phase consisted of acetonitrile and 5 mM ammonium formate with 0.1% formic acid in water (85:15, v/v), delivered at a flow rate of 0.25 mL/min, with an injection volume of 10 µL. Mass spectrometric detection was conducted in positive ion mode, monitoring the transitions m/z 336.4 → 321.4 for berberine (declustering potential (DP) of 63 V, collision energy (CE) of 32 V, and collision cell exit potential (CXP) of 10 V) and m/z 450.3 → 259.3 for crizotinib (DP 120, CE 30, and CXP 15). Crizotinib was selected as the IS due to its stable ionization, reproducible extraction, and lack of interference in the biological matrix.

PK analysis

Plasma concentrations of berberine are presented in ng/mL. The PK parameters were calculated using PKSolver, a Microsoft Excel (Microsoft Corp., Redmond, WA, USA)-based add-in that implements common PK and pharmacodynamic analyses such as non-compartmental analysis, compartmental model fitting (including special multiple-absorption and enterohepatic-circulation models), and a set of built-in PK functions [54]. The area under the plasma concentration-time curve (AUC) from time 0 to 8 h (AUC_0-8_) and 48 h (AUC_0-48_) was determined using the trapezoidal rule, applying either the linear or hybrid linear/logarithmic method depending on whether the concentration-time segment was ascending or descending. The terminal rate constant (λ_z_) was obtained by linear regression of the log-transformed concentration-time data for the terminal phase, and the apparent terminal half-life (t_1/2_) was calculated as ln_(2)_/λ_z_. The AUC extrapolated to infinity (AUC_0-∞_) was computed as the sum of AUC_0-48_ and the extrapolated portion (C_last/λ_z_). The area under the first moment curve (AUMC) was derived analogously by integrating t·C versus time, and the mean residence time (MRT) was calculated as AUMC/AUC, incorporating extrapolation beyond the last time point. The maximum observed concentration (C_max_) and its corresponding time (T_max_) were obtained directly from the experimental concentration-time data without interpolation. Relative bioavailability (F_rel_) was calculated using the formula

$F_{\mathrm{rel}} = \mathrm{AUC}_{\mathrm{test}}/\mathrm{AUC}_{\mathrm{reference}} (\mathrm{dose-normalized})$

Statistical analysis

Descriptive data are presented as mean ± standard deviation (SD). Statistical comparisons for C_max_ and AUC parameters (AUC_0-8_, AUC_0-48_, and AUC_0-∞_) were performed on log-transformed data using Welch’s t-test. Geometric mean ratios (GMRs) and corresponding 90% confidence intervals (CIs) were calculated by exponentiating the difference between group means and the associated CIs obtained on the log scale. T_max_ was summarized descriptively and not subjected to statistical comparison due to a lack of variability in the test group. The apparent terminal half-life (t_1/2_) and MRT were compared between groups using an independent samples t-test. A p-value of <0.05 was considered statistically significant. All statistical analyses were performed using JASP (Jeffreys’s Amazing Statistics Program; University of Amsterdam, Amsterdam, Netherlands).

## Results

**Figure 1:**
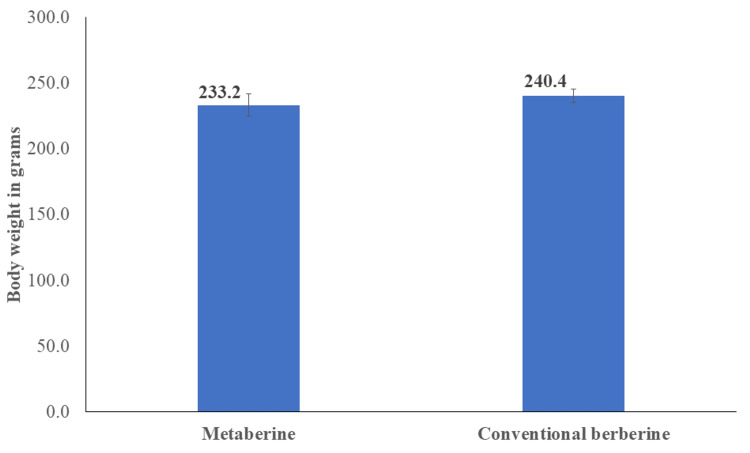
Body weight comparison of the test substance and conventional berberine groups

In aqueous media, the test substance produced rapid and uniform dispersion compared to the conventional berberine. No treatment-related mortality, morbidity, or abnormal clinical signs were observed in any of the animals during the experimental period in either group. Body weight profiles were comparable between groups throughout the study period, with no statistically significant differences. Mean (SD) body weight of animals in the test substance group was 233.2 (8.52) g with a range of 221.08 to 246.64 g, and the mean (SD) body weight of animals in the conventional berberine group was 240.4 (5.02) g with a range of 235.8 to 246.75 g (Figure 1).

The PK parameters of berberine following oral administration of the test substance and conventional berberine are summarized in Table 1.

**Table 1:** Pharmacokinetic (PK) parameters of berberine following oral administration of the test substance and conventional berberine (200 mg/kg body weight) Each value represents the mean ± SD. *p < 0.05 compared to conventional berberine. Statistical comparisons were for AUC values, and C_max_ were performed on log-transformed data using Welch’s t-test. T_max_ was not statistically compared due to zero variance in the test group. An independent samples t-test was performed for t_1/2_ and MRT. SD: standard deviation; AUC_0-8_: area under the plasma concentration-time curve from time 0 to 8 h; AUC_0-48_: area under the plasma concentration-time curve from time 0 to 48 h; AUC_0-∞_: area under the plasma concentration-time curve from time 0 to infinity; C_max_: peak plasma concentration; T_max_: time to reach C_max_; t_1/2_: apparent terminal half-life; MRT_(obs)_: observed mean residence time calculated as AUMC_0-48_/AUC_0-48_; AUC_0-8_ was included to assess early exposure due to differences in absorption rate.

PK parameters	Test substance	Conventional berberine
AUC_0-8_ (ng·h/mL)	177.7 ± 171.6*	68.28 ± 18.75
AUC_0-48_ (ng·h/mL)	257.73 ± 247.5	153.9 ± 30.48
AUC_0-∞_ (ng·h/mL)	261.74 ± 248.8	157.15 ± 31.36
C_max_ (ng/mL)	139.26 ± 182.3*	12.32 ± 4.12
T_max_ (h)	0.50 ± 0.00	8.00 ± 7.46
t_1/2_ (h)	8.63 ± 5.88*	3.64 ± 2.23
MRT_(obs)_ (h)	7.36 ± 1.70	8.81 ± 4.70

Enhanced systemic exposure

**Figure 2:**
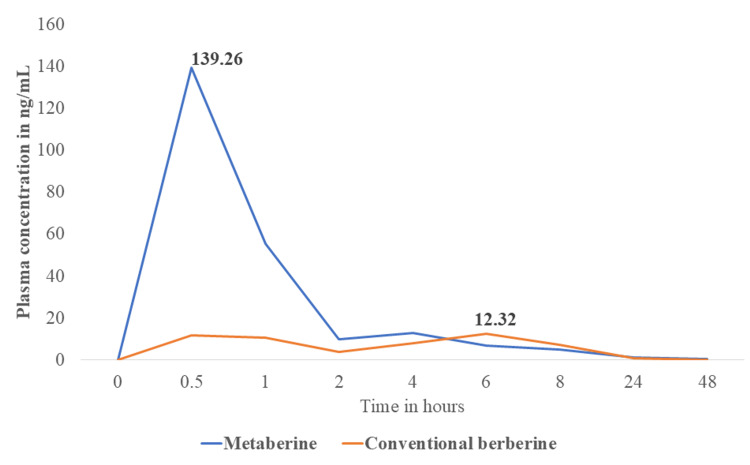
Plasma concentration-time curves of berberine following oral administration of the test substance and conventional berberine (200 mg/kg body weight) Values 139.26 and 12.32 represent the C_max_ of berberine from the test substance and conventional berberine, respectively.

The test formulation resulted in higher systemic exposure during the early phase. The mean AUC_0-8_ and AUC_0-48_ values were 177.7 and 257.73 ng·h/mL, respectively, compared to 68.28 and 153.9 ng·h/mL for conventional berberine, corresponding to approximately 2.6- and 1.7-fold higher exposure, respectively, when administered at the same dose of 200 mg/kg body weight. When normalized to the berberine active content, the test substance demonstrated 8.59-fold greater AUC_0-8_ and 5.5-fold greater AUC_0-48_ per mg/kg active compared with conventional berberine. Statistical analysis of log-transformed data showed a significant increase in AUC_0-8_ (p < 0.05), with a GMR of 2.07 (90% CI: 1.25-3.41). No statistically significant differences were observed for AUC_0-48_ or AUC_0-∞_, indicating comparable overall exposure between the formulations. The corresponding plasma concentration-time profiles are shown in Figure 2.

Higher peak plasma concentration (C_max_)

The mean C_max_ of berberine achieved with the test substance (139.26 ng/mL) was 11.3-fold higher than that of conventional berberine (12.32 ng/mL). When adjusted for the amount of active compound, the test substance exhibited a 37.3-fold higher C_max_ per mg/kg active than conventional berberine.

Earlier T_max _(systemic appearance)

Berberine from the test substance reached peak plasma levels within 0.5 h, compared with 8 h for conventional berberine, indicating an earlier T_max_ and more rapid systemic appearance. No statistical comparison was performed due to a lack of variability in the test group. 

Elimination parameters

The apparent terminal half-life (t_1/2_) of berberine from the test substance (8.63 h) was significantly higher in the test formulation compared to berberine from the conventional berberine (3.64 h) form, indicating a prolonged terminal phase in the concentration-time profile. No significant difference was observed in MRT between the groups (p > 0.05).

## Discussion

The present study compared the oral PK of a novel berberine formulation, Metaberine™, developed using proprietary (BioSOLVE) technology, with that of a conventional berberine in rats. The findings demonstrate that the test substance markedly enhanced peak plasma concentration and early systemic exposure relative to the conventional berberine.

The observed increases in AUC_0-8_ (2.6-fold) and C_max_ (11.3-fold) based on arithmetic means were supported by statistical analysis of log-transformed data. AUC_0-8_ showed a significant increase with a GMR of 2.07 (90% CI: 1.25-3.41), while C_max_ was significantly higher with a GMR of 6.46 (90% CI: 2.88-14.50). In contrast, AUC_0-48_ and AUC_0-∞_ did not differ significantly between the formulations, indicating that the enhancement was primarily driven by increased early systemic exposure rather than a marked increase in overall systemic exposure. The use of AUC_0-8_ as a partial AUC enables evaluation of early exposure, which is particularly relevant given the pronounced difference in T_max_ (0.5 h vs. 8 h) between the two formulations. When normalized to berberine active content, the test substance demonstrated 8.6-fold higher AUC_0-8_ and 5.5-fold higher AUC_0-48_ per mg/kg, along with a 37.3-fold higher C_max_ per mg/kg compared with conventional berberine. These findings highlight the efficiency of the formulation in delivering active berberine. The markedly earlier reduction in T_max_ indicates a more rapid systemic appearance of berberine from the test formulation. Such rapid systemic appearance may be relevant for applications where a rapid onset of systemic availability is desirable. However, as the earliest sampling time point was 0.5 h, T_max_ should be interpreted with caution, and the true peak may have occurred earlier. Considerable inter-animal variability was observed, particularly for C_max_, which is common in oral PK studies of berberine and related poorly bioavailable compounds.

The test substance also exhibited a prolonged apparent terminal half-life (t_1/2_ = 8.63 h) compared to conventional berberine (t_1/2_ = 3.64 h). However, given the lack of a significant difference in MRT, this observation should be interpreted with caution. The prolonged apparent terminal half-life (t_1/2_) may reflect altered absorption processes (e.g., flip-flop kinetics), wherein the terminal phase of the concentration-time profile can be influenced by the rate of absorption rather than true elimination. Conventional berberine undergoes extensive phase-I metabolism by CYP_450_ enzymes, yielding metabolites such as demethyleneberberine and berberrubine [55]. Additionally, gut microbiota contributes to its biotransformation, which, in combination with low absorption, results in very low plasma concentrations and rapid systemic clearance [27].

The observed PK characteristics are consistent with the design of the BioSOLVE technology system, which aims to improve apparent solubility through amphiphilic polysaccharide-based microencapsulation and fine colloidal dispersion. These characteristics may support improved interaction with the gastrointestinal environment and contribute to enhanced systemic availability of the encapsulated bioactive. Comprehensive physicochemical characterization of the formulation was beyond the scope of the present study, and further studies are warranted to better define the formulation properties contributing to the observed PK profile.

Despite its low oral bioavailability, berberine has demonstrated clinical efficacy, suggesting that its metabolites may substantially contribute to its pharmacological activity. Several metabolites exhibit independent biological effects. For example, berberrubine-9-O-β-D-glucuronide has shown glucose-lowering activity [25], berberrubine has been reported to demonstrate lipid-lowering [25] and anti-ulcerative colitis [25] effects, and demethyleneberberine has exhibited anti-inflammatory and hepatoprotective properties [25]. Reported PK studies further indicated that berberrubine-9-O-β-D-glucuronide achieved the highest systemic exposure (AUC_0-48_), followed by demethyleneberberine-2-O-β-D-glucuronide, thalifendine-10-O-β-D-glucuronide, jatrorrhizine-3-O-β-D-glucuronide, demethyleneberberine-2-O-sulfate, berberrubine, berberine, jatrorrhizine-3-O-sulfate, and demethyleneberberine in rats [25].

In the present study, only the parent compound was quantified, which is a commonly employed approach in comparative PK evaluations of formulation-dependent absorption. The observed increase in early systemic exposure of berberine with the test formulation, therefore, reflects enhanced systemic availability of the parent compound. While this may also influence metabolite exposure, conclusions regarding metabolite kinetics or overall pharmacological activity cannot be drawn from the current data. Future investigations incorporating metabolite profiling would provide a more comprehensive understanding of berberine disposition following administration of advanced formulations. No treatment-related clinical signs or adverse effects were observed in any of the animals, suggesting that the formulation was well tolerated at the administered dose.

These findings may have translational relevance, as conventional berberine supplementation typically requires relatively high daily doses (0.5-1.5 g/day) to achieve measurable clinical effects [5,13,22]. High-dose berberine is frequently associated with gastrointestinal discomfort [39], which may limit long-term adherence. Formulation strategies that enhance early systemic exposure and peak plasma concentration may potentially enable lower effective oral doses, which could improve tolerability and adherence; however, this requires confirmation in dedicated pharmacodynamic and clinical studies. Unlike many advanced delivery systems developed primarily for experimental purposes, the formulation evaluated here is intended for conventional oral administration using food-grade excipients. Nevertheless, direct comparisons with other enhanced formulations were beyond the scope of this study, and further research is required to determine relative performance across different technologies.

Conventional berberine required suspension in 0.5% CMC to facilitate administration, whereas the test formulation was readily dispersible in water due to its formulation characteristics. Accordingly, vehicle-related differences may have contributed to the observed PK profile and should be considered in the interpretation of the results. The dose administered to rats (200 mg/kg, formulation weight) was selected to achieve quantifiable plasma concentrations and enable reliable PK characterization, given the low oral bioavailability of berberine. The primary objective was to compare relative systemic exposure between formulations following oral administration rather than to establish inter-species dose equivalence or predict human dosing. Similar dose selection strategies are commonly used in preclinical PK studies of poorly bioavailable compounds [56]. It is acknowledged that this dose level may not directly translate to human use; however, the observed differences in PK parameters, particularly early systemic exposure and peak plasma concentration, are indicative of formulation-dependent improvements in systemic availability, which may be expected to persist across different dose levels. Further studies at clinically relevant doses are warranted to evaluate translational applicability.

This study focused on comparative PK evaluation and did not assess pharmacodynamic effects, tissue distribution, intestinal permeability, or transporter interactions. Consequently, while relatively increased systemic exposure indicates improved apparent availability, the specific mechanisms underlying this enhancement cannot be determined. Multiple processes, including improved dissolution, altered intestinal permeability, reduced efflux, or changes in presystemic metabolism, may contribute. Further mechanistic studies are required to elucidate the pathways involved. To facilitate approximate comparison of systemic exposure relative to the amount of active compound delivered, PK parameters were additionally expressed per unit dose of berberine; however, such normalization cannot fully account for potential matrix effects, excipient-mediated absorption changes, or differences in vehicle systems.

The strengths of the present study include the direct comparative PK evaluation using validated LC-MS/MS quantification and comprehensive non-compartmental analysis. In addition, the inclusion of multiple PK endpoints and prolonged plasma sampling up to 48 h provided a broad assessment of formulation-dependent PK behavior. Key limitations, including the absence of metabolite profiling, mechanistic characterization, pharmacodynamic assessment, and the potential influence of vehicle differences and inter-animal variability, are acknowledged throughout the Discussion section.

## Conclusions

In summary, Metaberine™ demonstrated improved PK performance compared to conventional berberine, characterized by enhanced early systemic exposure, markedly higher peak plasma concentration, and an increased apparent terminal half-life following oral administration at a dose of 200 mg/kg body weight in rats. These findings suggest that BioSOLVE technology may help address key biopharmaceutical limitations of berberine related to solubility and permeability. Further clinical PK and efficacy studies in humans are warranted to confirm these findings and to evaluate the potential of Metaberine™ as an advanced, low-dose, and well-tolerated berberine formulation for metabolic and cardiovascular health support.
